# The Effect of Poly(ethylene glycol) (PEG) Length on the Wettability and Surface Chemistry of PEG-Fluoroalkyl-Modified Polystyrene Diblock Copolymers and Their Two-Layer Films with Elastomer Matrix

**DOI:** 10.3390/polym12061236

**Published:** 2020-05-29

**Authors:** Elisa Guazzelli, Giancarlo Galli, Elisa Martinelli

**Affiliations:** Dipartimento di Chimica e Chimica Industriale and UdR Pisa INSTM, Università di Pisa, via Moruzzi 13, 56124 Pisa, Italy; elisa.guazzelli@dcci.unipi.it (E.G.); giancarlo.galli@unipi.it (G.G.)

**Keywords:** contraphilic wetting, amphiphilic polymer, block copolymer, SEBS, two-layer film, elastomer

## Abstract

Diblock copolymers composed of a polystyrene first block and a PEG-fluoroalkyl chain-modified polystyrene second block were synthesized by controlled atom transfer radical polymerization (ATRP), starting from the same polystyrene macroinitiator. The wettability of the polymer film surfaces was investigated by measurements of static and dynamic contact angles. An increase in advancing water contact angle was evident for all the films after immersion in water for short times (10 and 1000 s), consistent with an unusual contraphilic switch of the PEG-fluoroalkyl side chains. Such a contraphilic response also accounted for the retained wettability of the polymer films upon prolonged contact with water, without an anticipated increase in the hydrophilic character. The copolymers were then used as surface-active modifiers of elastomer poly(styrene-*b*-(ethylene-*co*-butylene)-*b*-styrene) (SEBS)-based two-layer films. The elastomeric behavior of the films was varied by using SEBS matrices with different amounts of polystyrene. Whereas the mechanical properties strictly resembled those of the nature of the SEBS matrix, the surface properties were imposed by the additive. The contraphilic switch of the PEG-fluoroalkyl side chains resulted in an exceptionally high enrichment in fluorine of the film surface after immersion in water for seven days.

## 1. Introduction

Polymer surface-active additives are used to modify and opportunely modulate the surface properties of the polymer matrix into which they are incorporated—either physically dispersed [1,2,3,4,5,6,7,8] or chemically linked [9,10,11,12,13,14,15]. In fact, by taking advantage of the ability of the additive to selectively segregate at the surface of a polymer film, the wettability properties are commonly changed [16,17,18,19]. Several factors are known to affect the surface segregation of the polymer additive, including its molar mass, chemical structure, composition, surface tension and chemical compatibility with the host matrix [20,21,22,23,24]. Moreover, surface segregation being a process influenced by the nature of the external environment (e.g., air, water, oil, organic vapors, biological fluids) the additive’s sensitivity to the external environment also plays a role [25,26,27,28]. Polysiloxane-based surface-active additives are generally used to enhance the surface hydrophobicity and photooxidation resistance, while perfluoroalkyl-based additives are known to impart both high hydrophobicity and lipophobicity, resulting in slippery [29] and self-polishing properties [30]. On the other hand, polyethylene glycol-based surface-active additives are exploited to promote a non-ionic hydrophilic behavior in a polymer film surface, and improve its antifouling properties against proteins, bacteria, cells and marine organisms [31,32,33]. 

The use of amphiphilic polymer surface active-additives, derived from the combination of hydrophilic and hydrophobic components in the same macromolecular structure, has gained interest in recent decades as a straightforward and useful strategy to tailor surface- and interface-structuring factors of wettability, reconstruction, morphology and topography at the nanoscale of polymer films [17,18,34,35]. In particular, amphiphilic surface-active additives, composed of polysiloxane and/or fluoropolymer chains as the hydrophobic components, and poly(ethylene glycol) (PEG) chains as the hydrophilic components, have been investigated in different fields of nanotechnology [3,34,36,37,38,39]. The self-assembly of such amphiphilic copolymers in fact is also possible in the bulk copolymer itself [40,41], or when dissolved in a selective solvent, determining a variety of nanostructures ranging from single chain nanoparticles to large aggregates [42,43,44,45,46]. A surface reconstruction process of amphiphilic polymers generally involves an increase in hydrophilicity of the film surface after immersion in polar solvents, notably water, and a reversible restoration of the surface hydrophobicity after contact with air [4,17]. The surface wettability of amphiphilic PEG-fluoroalkyl polymer films with relatively short PEG segments (~5 CH_2_CH_2_O groups) was found to drastically increase upon immersion in water [6]. However, a counterintuitive response to the polar environment, which involved an increase in surface hydrophobicity after immersion in water, was also reported for a few examples, including fluorous-based [47,48,49,50] and fluorous-free amphiphilic polymers [51,52]. This unusual response to the water environment is generally referred to as contraphilic behavior, as opposed to the amphiphilic wetting typical of hydrophilic/hydrophobic, i.e., amphiphilic, systems.

By taking advantage of the controlled nature of atom transfer radical polymerization (ATRP), we synthesized amphiphilic diblock copolymers, composed of a polystyrene first block and a polystyrene second block modified with a mixed PEG-fluoroalkyl side chain consisting of ~12 CH_2_CH_2_O and ~4 CF_2_CF_2_ groups. The length of the PEG segment, as well as the degree of polymerization of the second block, were varied for a tuning of the hydrophobic/hydrophilic balance of the diblock copolymer, in such a way that an environment-induced contraphilic response could be effected. The measurements of the dynamic and static contact angles of the films therefrom evidenced a contraphilic behavior, with a significant increase in water advancing contact angle and a retention of the wettability properties of the polymer surfaces, even after a relatively prolonged period of immersion in water. The copolymers were then used as non-reactive, physically dispersed surface-active additives to prepare elastomeric, SEBS-based two-layer films. It was found that the films displayed an exceptionally high surface enrichment in fluorine after immersion in water, as a result of the surface-active additive contraphilic behavior, along with an elastomeric character typical of the SEBS matrix. The combined features of low bulk elastic modulus and contraphilic surface properties of these films may be exploited for the development of biocide-free, soft coatings with a dynamically (nano) restructuring surface to combat marine biofouling, as novel alternative candidates to the closely related and widely investigated amphiphilic fouling-release coatings [8,24,34,35,53,54,55,56]. Such films are expected to perform against organisms, in fact showing different adhesion profiles and contrasting preferences to hydrophilic, or otherwise hydrophobic, surfaces.

## 2. Materials and Methods

### 2.1. Materials

Anisole was kept at 100 °C over sodium for 4 h and then distilled under reduced pressure. 2,2′-Bipyridine (Bipy), CuBr and 1-phenylethyl bromide (1-(PE)Br) were purchased from Sigma-Aldrich (St. Louis MO, USA) and used without further purifications.

The monomer SzA was synthesized according to the procedure previously reported from 4-vinylbenzoic acid (from Sigma-Aldrich, St. Louis MO, USA) and the PEG-fluoroalkyl alcohol (Zonyl FSN-100 F(CF_2_CF_2_)_y_(CH_2_CH_2_O)_x_CH_2_CH_2_OH, x = 0–25 and y = 1–9) (registered trademark of E. I. du Pont de Nemours & Co) (from Sigma Aldrich, St. Louis MO, USA), respectively [6]. According to size exclusion chromatography (SEC) and nuclear magnetic resonance (NMR) measurements, the monomer SzA synthesized presented a relatively broad distribution of molecular weights (*Ð*~1.3) and average degrees of polymerization y~4 and x~12.

Styrene (S) (from Fluka) was washed with 5% NaOH and water, dried over Na_2_SO_4_ and distilled under reduced pressure prior to use. (3-Glycidyloxypropyl)-trimethoxysilane (GPS) (from Gelest, Morrisville PA, USA) was used without further purification. Poly(styrene-*b*-(ethylene-*co*-butylene)-*b*-styrene) (SEBS) triblock thermoplastic elastomer [Kraton G1657M and Kraton 1652M containing 13 wt % (E13) and 30 wt % (E30) polystyrene, respectively] and SEBS grafted with maleic anhydride (MA) [Kraton FG1924X with 0.7–1.3 wt % MA and 13 wt % polystyrene (E13-MA) and Kraton FG1901X with 1.4–2.0 wt % MA and 30 wt % polystyrene (E30-MA)] were kindly provided by Kraton Corporation (Wesseling, Germany).

### 2.2. Polymer Synthesis

**Figure 1:**
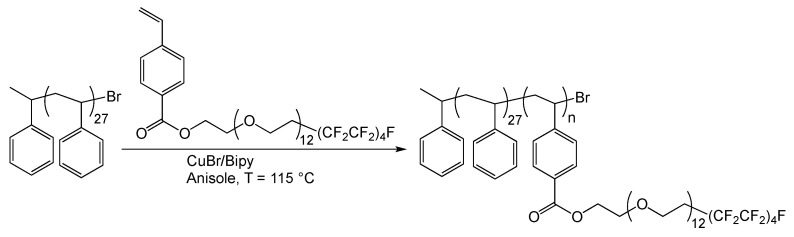
Schematic of the atom transfer radical polymerization (ATRP) synthesis of the diblock copolymers S27SzAn by macroinitiator S27.

The synthetic procedure is illustrated in Figure 1.

#### 2.2.1. Synthesis of Polystyrene Macroinitiator

50.00 mL (0.437 mol) of S, 3.427 g (21.53 mmol) of Bipy and 990 μL (7.24 mmol) of 1-(PE)Br were introduced into a dry Schlenk flask under nitrogen. The solution was purged with nitrogen for 15 min and then 1.036 g (7.22 mmol) of CuBr was added. After three freeze–thaw pump cycles, the polymerization was allowed to proceed under nitrogen for 90 min at 110 °C. When the reaction was stopped the polymer solution was diluted with tetrahydrofuran and then eluted on neutral alumina to remove the catalyst. The solvent was removed under vacuum and the polymer was purified by repeated precipitations from tetrahydrofuran solutions into methanol (48% yield). The number average degree of polymerization of the polymer sample was 27, and the macroinitiator is named here S27.

^1^H NMR (CDCl_3_): *δ* (ppm) = 0.8–2.4 (CH_2_CH), 6.2–7.4 (aromatic).

FT-IR (film): *ν* (cm^–1^) = 3082–3026 (*ν* C–H aromatic), 2924 (*ν* C–H aliphatic), 1601 (*ν* C=C aromatic), 1493–1453 (*δ* C–H aliphatic), 757 and 698 (*δ* C–H aromatic).

#### 2.2.2. Synthesis of S27SzAn Diblock Copolymers

In a typical preparation, 0.853 g (0.30 mmol) of S27 and 0.143 g (0.91 mmol) of Bipy were introduced into a dry Schlenk flask, which was then evacuated and flushed with nitrogen three times. A solution of 6.511 g (6.03 mmol) of SzA in 30 mL of anisole was then added under nitrogen. The mixture was purged with nitrogen for 30 min and then 0.046 g (0.322 mmol) of CuBr was added. After four freeze–thaw pump cycles, the polymerization was let to proceed for 66 h at 115 °C. When the reaction was stopped, the polymer mixture was dissolved in dichloromethane and then repeatedly washed with water until discoloration of the water. The solvent was removed under vacuum and the polymer was purified by repeated cold extractions with methanol (44% yield). The number average degrees of polymerization of the S and SzA blocks were 27 and 11, respectively, and the block copolymer is named here S27SzA11. 

^1^H NMR (CDCl_3_): *δ* (ppm): 0.9–2.0 (CH_2_CH), 2.4 (CH_2_CF_2_), 2.9–4.1 (CH_2_O), 4.4 (COOCH_2_), 6.0–8.2 (aromatic).

^19^F NMR (CDCl_3_/CF_3_COOH): *δ* (ppm): –6 (CF_3_), –38 (CF_2_CH_2_), –46 to –49 (CF_2_), –51 (2F, CF_2_CF_3_).

FT-IR (film): *ν* (cm^–1^) = 3060–3024 (*ν* C–H aromatic), 2916 (*ν* C–H aliphatic), 1716 (*ν* C=O), 1609 (*ν* C=C aromatic), 1493 e 1454 (δ C−H aliphatic), 1400–1000 (*ν* C–O and *ν* C–F), 655 (*ω* CF_2_).

### 2.3. Deposition of One-Layer Films

One-layer films were prepared by spin- or dip-coating. For spin-coating technique, a 3 wt% solution of the neat block copolymer in CHCl_3_ was filtered on PTFE filter (0.2 μm) and spin-coated (5000 rpm for 15 s) on glass supports. For dip-coating technique, glass cover slips, pre-treated with chlorotrimethylsilane, were dipped in a filtered 0.5% *w*/*v* solution of the neat block copolymer in toluene. The films were then vacuum-dried at 120 °C overnight and annealed in the oven at 120 °C for 12 h. 

### 2.4. Deposition of SEBS-Based Two-Layer Films

The detailed procedure followed for the preparation of two-layer films was previously described [7]. Briefly, GPS-functionalized glass slides were coated by casting on a 12% *w*/*v* toluene solution of a blend of SEBS (E30 and E13) (44 wt %) and the corresponding SEBS-MA (E30-MA and E13-MA) (56 wt %). The films were allowed to dry slowly in a closed chamber for three days and then annealed in an oven overnight at 120 °C to form the bottom layer. A 1.5% *w*/*v* toluene solution of either a block copolymer alone or a blend of a block copolymer with SEBS was spray-coated on the bottom layer using a Badger model 250 airbrush (50 psi air pressure). The films were vacuum-dried in an oven at 60 °C for 8 h and then annealed at 120 °C overnight to form the top layer. Accordingly, the films are denoted as E13-S27SzAn_z or E30-S27SzAn_z, where n indicates the degree of polymerization of the block SzA, and z is the weight percentage of copolymer in the blend with SEBS in the top layer. 

Two-layer films not containing the copolymer in the top layer were also prepared for comparison, by spray-coating the corresponding SEBS copolymer alone on the bottom layer. 

### 2.5. Characterization

^1^H NMR (vs. TMS) and ^19^F NMR (vs. CF_3_COOH) spectra were recorded on Varian Gemini VRX 200 and Varian Gemini VRX 300 spectrometers (Palo Alto, CA, USA), respectively. Infrared spectra were recorded with a Spectrum One Perkin-Elmer Fourier Transform infrared spectrophotometer with 4 cm^–1^ resolution. Polymer films were cast on a KBr crystal plate. 

The number and weight average molecular weights of the polymers, *M*_n_ and *M*_w_ respectively, and dispersity (*Ð*) were determined by size exclusion chromatography (SEC) with a Jasco PU-1580 liquid chromatograph (Hachioji-shi, Tokyo, Japan) equipped with two PL gel 5 μm Mixed-D columns, a Jasco 830-RI refractive index detector and a Perkin-Elmer LC75 UV detector. Polystyrene standards (4.0 × 10^2^–4.0 × 10^5^ g/mol) were used for calibration. 

Contact angles were measured by the sessile drop method with a FTA200 Camtel goniometer (Portsmouth, VA, USA), using water (*θ*_w_) (J. T. Baker, HPLC grade) and *n*-hexadecane (*θ*_h_) (from Aldrich of the highest purity available) as wetting liquids. The measured values of *θ*_w_ and *θ*_h_ were used to extract the surface tension (***γ***_s_^OWK^) of the polymer films according to the so-called Owens–Wendt–Kaelble method [57,58]. 

Water advancing and receding (*θ*_a_, *θ*_r_) contact angle experiments were carried out on one-layer films by the Wilhelmy balance method using a Dataphysics dynamic contact angle tensiometer DCAT-11 with immersion–withdrawal rates of 200 μm/s. Two different experiments were performed: (i) three immersion cycles at 8 mm immersion depth, with dwell times between immersion and withdrawal of 10 s; (ii) one immersion cycle at 8 mm immersion depth with a dwell time of 1000 s, followed by an immersion cycle at 14 mm immersion depth, so that an additional 6 mm of fresh surface was exposed to water, and a dwell time of 10 s.

X-ray photoelectron spectroscopy (XPS) spectra were recorded using a Perkin-Elmer PHI 5600 spectrometer (Chanhassen, MN, USA) with a standard Al-Kα source (1486.6 eV) operating at 350 W. The working pressure was less than 10^−8^ Pa. The spectrometer was calibrated by assuming the binding energy (BE) of the Au 4f_7/2_ line to be 84.0 eV with respect to the Fermi level. Extended spectra (survey) were collected in the range 0−1350 eV (187.85 eV pass energy, 0.5 eV step, 0.025 s/step). Detailed spectra were recorded for the following regions: C(1s), O(1s) and F(1s) (11.75 eV pass energy, 0.1 eV step, 0.1 eV s/step). The spectra were recorded at the three photoemission angles *φ* (between the surface normal and the path taken by the electrons to the detector) of 70° and 20°, corresponding to sampling depths of ca. 3–8 nm. The standard deviation in the BE values of the XPS line was 0.10 eV. The software used for background subtraction (Shirley type) [59] and quantitative analysis was the PHI software (version 5.2) for data collection in PHI 5600ci Multitechnique (Chanhassen, MN, USA). The atomic percentage was evaluated using the PHI sensitivity factors [60]. To take into account charging problems, the C(1s) peak was considered at 285.0 eV and the peak BE differences were evaluated. The XPS peak fitting procedure was carried out, after a Shirley type background subtraction, by means of Voigt functions and the results evaluated through the χ^2^ function [61].

Scanning electron microscope (SEM) images were recorded with an electronic microscope JEOL 5600 LV operating at 13 kV and 20 kV. Before observation, the film was fractured in liquid nitrogen and then vacuum-metallized.

Tensile stress-strain experiments were performed at ambient temperature using a 5564 Instron machine (Instron, High Wycombe, UK). Samples were cut into microtensile test specimens and pre-conditioned for 3 days at 25 °C and 50% relative humidity in a chamber containing a saturated solution of magnesium nitrate. Testing protocols were based on American Society for Testing and Materials Standard test D1708-73 and D412-92. Crosshead speed was set at 254 mm/min. 

## 3. Results and Discussion

### 3.1. Synthesis of Block Copolymers

Diblock copolymers S27SzAn were synthesized according to a two-step procedure reported in [6]. First, a bromo-terminated polystyrene macroinitiator, S27 (*M*_n_ = 2800 g/mol, number average degree of polymerization 27), was prepared by ATRP of styrene, S, using 1-phenylethyl bromide (1-(PE)Br)/CuBr/2,2’-bipyridine (Bipy) (1/1/3 mole ratio) at 110 °C for 90 min. Then, the obtained bromo-terminated polystyrene was used as a macroinitiator to initiate the polymerization of the amphiphilic monomer SzA at 115 °C in anisole solution. Different lengths of the SzA second block were obtained by varying the initial monomer SzA/macroinitiator mole ratio in the range 5–56, while keeping constant the CuBr/macroinitiator mole ratio (1/1) and the reaction time (66 h) (Figure 1, Table 1). 

^19^F NMR (Figure S1, in Supplementary Materials) and ^1^H NMR spectroscopies confirmed the incorporation of the SzA second block to the polystyrene first block. The chemical composition of the copolymers was evaluated from the integrated areas of the ^1^H NMR signals at 4.4 ppm (COOCH_2_ protons of SzA) and 6.0–8.2 ppm (aromatic protons of S). Generally, the SEC curves of the block copolymers were monomodal and characterized by a dispersity 1.3 ≤ *Ð ≤* 1.5 (Figure S2).

### 3.2. Wettability of Block Copolymer Films

The wettability of amphiphilic block copolymer films was investigated by measurement of both static and dynamic contact angles, in order to evaluate the surface stability when in contact with the interrogating liquids, in particular water, and its ability to reconstruct upon contact with water over different time scales. 

#### 3.2.1. Dynamic Contact Angles

**Figure 2:**
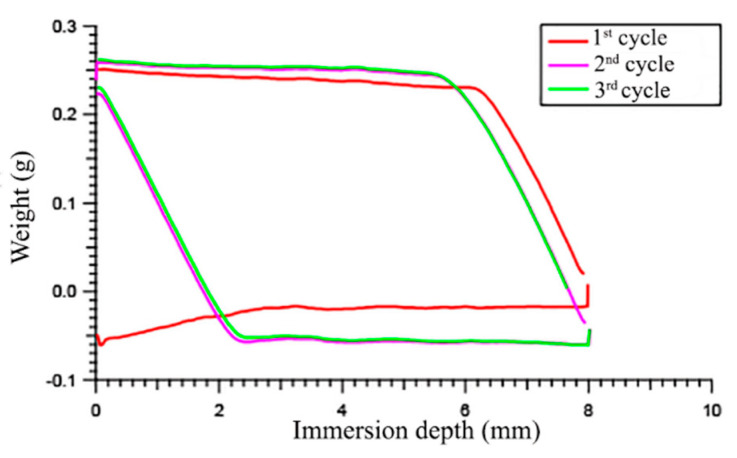
Wilhelmy plate force-immersion depth curves for the polymer S27SzA11: three cycles of immersion, 8 mm depth/10 s dwell time.

Two different experiments were carried out by applying different immersion cycles and dwell times. The results from the first experiment showed that the advancing angle *θ*_a_ was in the range 87°–96° for the first cycle, while it slightly increased to 90°–98° in the second and third cycles (Table 2). On the other hand, the receding angle *θ*_r_ was much lower, around 38°–65°, and was similar in all three cycles (Figure 2). As a result, the hysteresis (Δ = *θ*_a_ − *θ*_r_) was high in any case, and increased in the second and third cycles (Table 2).

**Figure 3:**
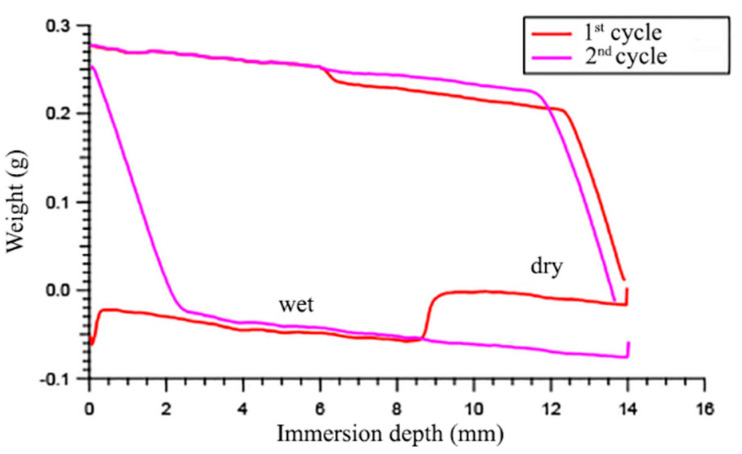
Wilhelmy plate force-immersion depth curves for the film S27SzA11: 1st cycle 8 mm depth/1000 s dwell time, and 2nd cycle 14 mm depth/10 s dwell time.

The increase in *θ*_a_ for the wet surface was more marked in the second experiment, where the *θ*_a_ of the first 8 mm of the second cycle (after 1000 s of immersion in water) was higher than the respective one for the second 6 mm when a fresh surface was exposed, thereby causing a net discontinuity in the graph (Figure 3). For example, in the case of S27Sz*A*11 film, *θ*_a_ = 88° for the fresh surface, and *θ*_a_ = 94° for the immersed surface (after 1000 s dwell time). The increase in *θ*_a_ of the wet surface suggests that the polymer surface became more hydrophobic after immersion, instead of more hydrophilic as would be typically expected. In fact, analogous amphiphilic block copolymers were previously found to exhibit decreased *θ*_a_ values in similar immersion experiments [6]. The increased hydrophobicity of the film surface after contact with water was not due to the leaching out of the physically dispersed surface-active additive, since the surface tension of the wetting water revealed no significant change with respect to the initial value of the neat water (72.75 mN/m at room temperature). Therefore, this peculiar wetting behavior was attributed to a contraphilic phenomenon. Only a few examples are reported in the literature on the increase in surface hydrophobicity induced by water [27,47,49,52], and some of them include the presence of fluorinated and oxyethylenic moieties in the same polymer structure [47,52]. In particular, Wynne et al. found out that fluorinated-oxyethylenic polyurethane surface modifiers displayed a typical amphiphilic, or alternatively a contraphilic, behavior by varying the length of the oxyethylenic side chain from 3 to 7 –CH_2_CH_2_O– units [47]. This is consistent with the contraphilic nature of the present copolymers with longer PEG segments, containing as many as 12 –CH_2_CH_2_O– units on average, as opposed to the amphiphilic wettability of chemically and structurally analogous copolymers carrying shorter PEG segments (5 –CH_2_CH_2_O– units on average) in the side chains [6]. Lower *θ*_a_ values for the dry surface with respect to the wet one are indicative of a lower concentration of fluorinated moieties at the polymer–air interface, possibly due to the formation of hydrogen bonds between the electron-poor –CH_2_CF_2_– hydrogens and the electron-rich –CO(O)– or –CH_2_CH_2_O– groups. Upon immersion in water, the formation of stronger hydrogen bonds of –CO(O)– or –CH_2_CH_2_O– with water causes a major exposure of the fluorinated groups to the polymer–water interface, thus producing a contraphilic effect. 

#### 3.2.2. Static Contact Angles

Contact angles of block copolymers with *n*-hexadecane (*θ*_h_) and water (*θ*_w_) were found to be in the ranges 64°–65° and 83°–85°, respectively, typical of lipophobic and moderately hydrophobic materials (Table 3). Analogous polystyrene-based block copolymers carrying shorter PEG segments in the amphiphilic side chains were reported to show higher *θ*_w_ and similar *θ*_h_ values [6]. This suggests that the longer PEG segments were more effectively located at the polymer film surface, thus increasing the surface hydrophilicity, without affecting the lipophobicity of the polymer films to a significant extent. The latter feature, in fact, appeared to be only dependent on the fluorinated segment.

**Figure 4:**
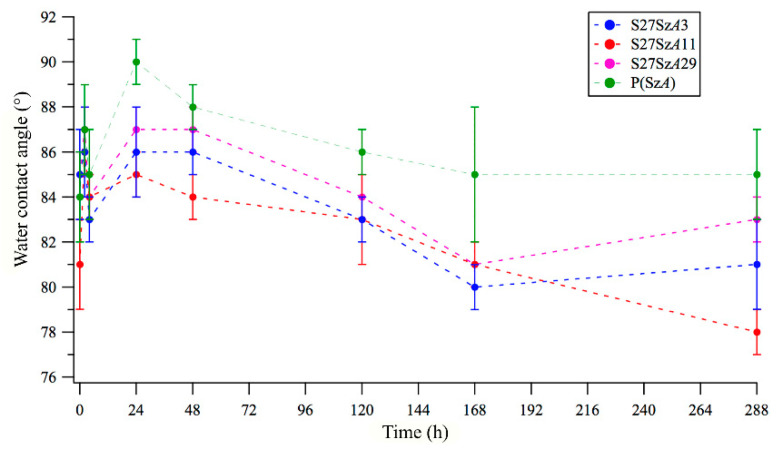
Water static contact angle as a function of immersion time in water.

Water static contact angles were also measured on amphiphilic polymer films as a function of immersion time in water up to 12 days (Figure 4). *θ*_w_ increased in the first 24 h of immersion by about 3°–4° for all the tested samples, consistent with a contraphilic behavior as discussed above. However, after the first day of immersion, *θ*_w_ decreased very slightly, reaching a value not significantly different from the initial one after 12 days. This trend suggests a water-driven reconstruction according to concomitant mechanisms characterized by different kinetics. Specifically, the contraphilic switching of the fluorinated side chain is fast and occurs over short immersion times (seconds according to dynamic contact angle). On the other hand, hydrophilic oxyethylenic segments placed very close to the polymer surface become more exposed to the polymer–water interface upon longer immersion times, thus contrasting at least partially with the contraphilic behavior of the entire system. In fact, as a result of the contraphilic switch of the side chains, oxyethylenic segments were dragged to the polymer surface by the covalently linked fluorinated tails. This migration process was also favored by the high flexibility of the long, hydrophilic oxyethylenic chain segments. This hypothesis was confirmed by XPS results, as discussed below. Overall, the reconstruction process upon the investigated immersion time of 12 days did not result in a significant increase in film surface hydrophilicity. This was in contrast with what was previously found for similar copolymers with shorter PEG segments in the side chains, which displayed a drastic reduction in water contact angle by ~30° upon immersion in water for 14 days [6]. 

### 3.3. Preparation of Two-Layer Films

S27SzAn block copolymers were used as non-covalently linked surface-active additives to prepare films with a two-layer geometry. This strategy was exploited to combine the surface properties imparted by the additive with the low elastic modulus of the matrix [5,17]. Films were prepared according to an established procedure [7]. Specifically, a toluene solution of SEBS/SEBS-MA was cast on glass slides previously cleaned in a hot piranha solution (H_2_O_2_/H_2_SO_4_ 70/30 v/v) and then immersed in a 2 wt % ethanol solution of (3-glycidyloxypropyl)-trimethoxysilane (GPS) in order to graft highly reactive glycidyl groups on the glass surface. Upon reaction of such glycidyl groups with the maleic anhydride groups of SEBS-MA, covalent anchoring of the SEBS/SEBS-MA film to the glass substrate was achieved (Figure S3). Then, a second layer of the block copolymer, alone or blended (10 wt %) with SEBS, was spray-coated on the top of the SEBS/SEBS-MA bottom layer to obtain two-layer films (overall thickness of ~250 μm). In order to modulate the elastomeric properties of the two-layer films, two types of SEBS/SEBS-MA systems, namely E13/E13-MA and E30/E30-MA, with 13 and 30 wt % polystyrene, respectively, were chosen to constitute the bottom layer. 

**Figure 5:**
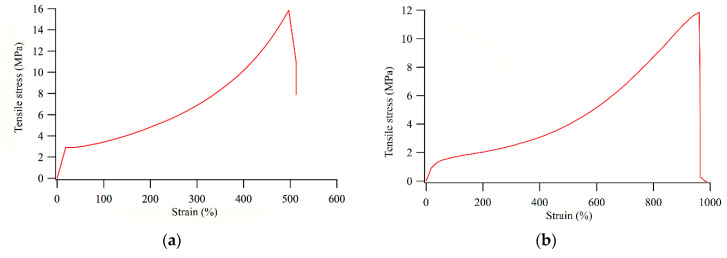
Stress-strain curves for E30-S27SzA3_100 (**a**) and E13-S27SzA3_100 (**b**).

Stress (*σ*)-strain (*ε*) mechanical tests were performed to evaluate the tensile properties of the films. Films based on the two different bottom layers presented *σ*-*ε* curves with a similar shape, apart from the initial linear region, which was more defined for the E30/E30-MA-based films, characterized by a higher elastic modulus (*E* = 3.6 ± 0.5 MPa) (Figure 5). On the other hand, E13/E13-MA-based films displayed a halved elastic modulus value (*E* = 1.9 ± 0.0 MPa) as well as lower stress at break and higher elongation at break (Table 4). These results are consistent with a more elastomeric behavior of the E13/E13-MA-based films, containing a lower amount of polystyrene block in the SEBS copolymer. For both E30 and E13-based two-layer films, the values of *E*, *σ* and *ε* appeared not to be significantly affected by the addition of the amphiphilic block copolymer to the top layer (Table 4). This finding is in agreement with what was previously reported for other SEBS-based amphiphilic films [5].

**Figure 6:**
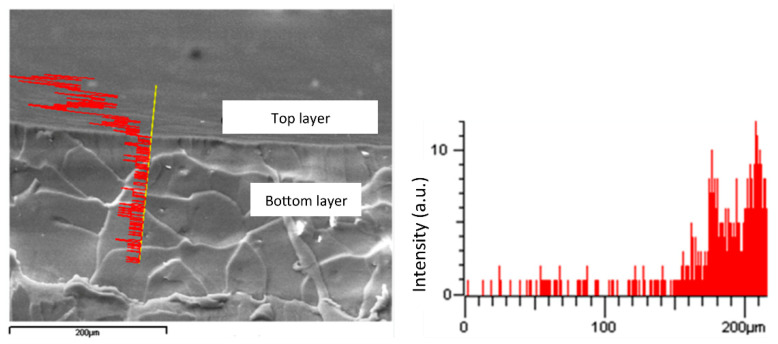
Energy dispersive X-ray EDX analysis of a cryogenic fracture section of a two-layer film (red line indicates fluorine concentration).

SEM images on cryogenic fracture sections proved that the two layers actually adhered well to each other, thanks to the compatibilizing role of the polystyrene block of the copolymers. Moreover, energy dispersive X-ray EDX analysis revealed that the fluorinated block copolymer was actually confined to the top layer, without migrating significantly inward to the SEBS bottom layer (Figure 6).

### 3.4. Wettability and Surface Tension of Two-Layer Films

The static contact angles of two-layer films were determined using two wetting liquids; water and *n*-hexadecane (Table 5). The water static contact angle (*θ*_w_) was generally around 100°, and higher than that of the corresponding pristine block copolymer, being, for example, 104° for the two-layer film E13-S27SzA29_100, and 83° for the respective block copolymer S27SzA29. This increase in *θ*_w_ suggests a SEBS-driven microphase separation, which enhanced the chemical incompatibility between the polystyrene block and the amphiphilic fluorinated polystyrene block of the copolymer. The values of *θ*_h_ for the two-layer films were similar to those of the corresponding pristine block copolymers, and confirmed the presence of the low surface energy fluorinated segments at the polymer–air interface, since they were much higher (*θ*_h_ = 63°–67°) than the values measured for the unmodified SEBS matrices (*θ*_h_ = 28°–39°). However, no trend of *θ*_w_ and *θ*_h_ variation was identified with the copolymer chemical composition and its amount in the top layer. Such hydrophobic and lipophobic behavior resulted in a generally low surface energy of the two-layer films, with ***γ***_s_^OWK^ of between 16.0 and 18.9 mN/m, consistent with a fluoroalkyl-populated surface due to the preferential surface segregation. These ***γ***_s_^OWK^ results were also significantly higher than those typical of a non-fluorinated hydrocarbon surface (***γ***_s_^OWK^ = 25.5 and 23.4 mN/m for E30 and E13, respectively).

### 3.5. Surface Composition of Two-Layer Films

Atomic surface compositions of the two-layer films were determined at different photoemission angles, *φ* = 70°, 50° and 20°, by angle-resolved XPS. Experimental data for the films are collected in Table 6, where they are also compared with the theoretical composition of the corresponding block copolymers. These values represent the theoretical upper limits in fluorine and oxygen concentration for all the tested films.

**Figure 7:**
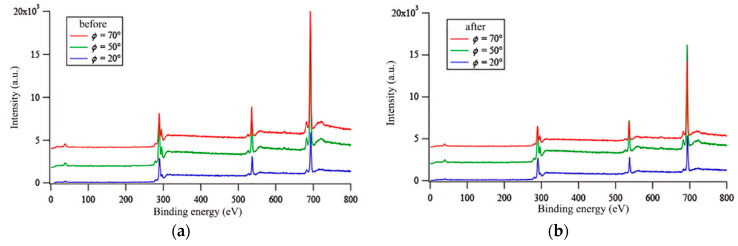
X-ray photoelectron spectroscopy (XPS) survey spectra for E13-S27Sz*A*11_100 at three different photoemission angles before (**a**) and after (**b**) 7 days of immersion in water.

Only the signals of the elements, C(1s)~290 eV, O(1s)~533 eV and F(1s)~689 eV, constituting the repeat units, were detected in the survey spectra recorded at any photoemission angle (Figure 7a). 

At any *φ*, the atomic percentage of fluorine at the surface was much higher than the theoretical value calculated for the corresponding block copolymer incorporated in the top layer. Moreover, there was a significant composition gradient along the polymer surface, and the fluorine concentration was markedly reduced, passing from *φ* = 70° to 20°. For example, the F percentage decreased from 39.5% (*φ* = 70°) to 33.6% (*φ* = 50°), down to 24.7% (*φ* = 20°) for the film E13-S27Sz*A*11_100. On the other hand, the C percentage increased from 47.9% (*φ* = 70°) to 60.0% (*φ* = 20°), thus tending to the theoretical value (67%) by increasing the sampling depth. The O percentage showed a similar, but much less marked, trend by increasing from 12.6% (*φ* = 70°) to 13.6% (*φ* = 50°), up to 15.3% (*φ* = 20°), the latter value corresponding to the theoretical one (15%). The high enrichment in fluorine of the film surfaces was independent of the chemical composition of the block copolymer, as well as its amount in the top layer. Moreover, all the tested films exhibited comparable surface compositions with each other, suggesting that a threshold in the nominal fluorine concentration exists, above which the surface is no more enriched in fluorine.

An angle-resolved XPS analysis was also carried out on the films after immersion in water for 7 days, with the aim of highlighting water-driven surface modification phenomena. The atomic compositions of the film surfaces after immersion in water are also collected in Table 6. All the aforementioned remarks for the surface before immersion are also valid for the surface after relatively prolonged immersion in water (Figure 7b, Table 6). It is noteworthy that the films after immersion exhibited a surface composition very close to the one they showed in the dry state. It is especially relevant that the fluorine percentage at *φ* = 70° exhibited a tendency to increase after immersion in water. Although such an increase was slight, it was consistent with the contraphilic wetting of the pristine block copolymers detected by dynamic contact angle measurements. The film surfaces did not undergo fluorine depletion, thus maintaining their hydrophobic nature. 

**Figure 8:**
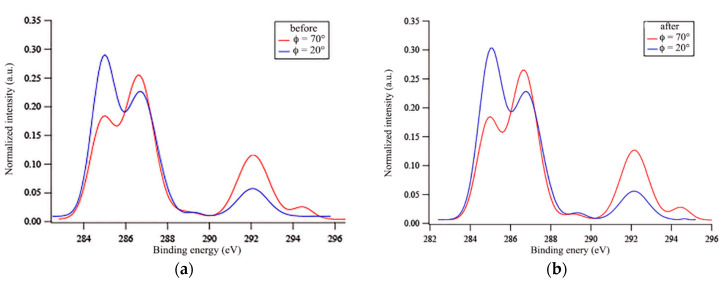
Area-normalized C(1s) XPS signals at *φ* of 70° and 20° for the film E13-S27Sz*A*11_100 before (**a**) and after (**b**) 7 days of immersion in water.

**Figure 9:**
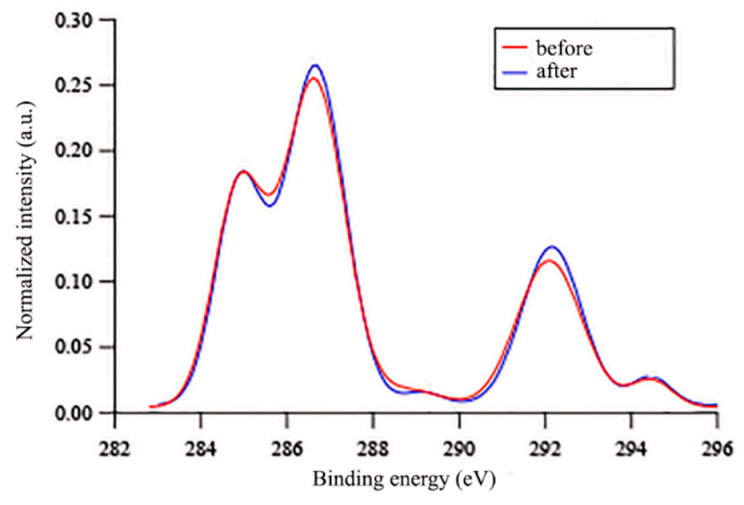
Area-normalized C(1s) XPS signals at *φ* = 70° for the film E13-S27Sz*A*11_100 before and after 7 days of immersion in water.

In any case, the C(1s) peak showed a complex shape due to the presence of at least five overlapping contributions at ~285 eV (CH, CH_2_ and C=C), ~287 eV (CH_2_CF_2_ and CH_2_O), ~289 eV (COO), ~292 eV (CF_2_), and ~294 eV (CF_3_) (Figure S4). From the comparison of the area-normalized C(1s) spectra at *φ* of 70° and 20°, it was evident that the contributions due to CF_2_ and CF_3_ groups drastically decreased with decreasing *φ* (Figure 8a). Interestingly, the intensity of the peak at~287 eV, mainly due to CH_2_O groups, also decreased with *φ,* that is, with increasing sampling depth (Figure 8a). The fluorinated and oxyethylenic groups also followed the same trend regarding *φ* after immersion in water (Figure 8b), suggesting that both the PEG and fluoroalkyl segments of the side chains preferentially populated the film surface, rather than the bulk, when in contact with either air or water. By a comparison of the C(1s) signal at *φ* = 70° for the same film before and after immersion in water (Figure 9), a slight increase in the intensities of peaks, due to both CH_2_O and CF_2_ groups, was noticed. This result is consistent with the two-step surface reconstruction mechanism previously discussed, in which a first quick surface modification involves the contraphilic switch of the side chains with a major exposure of the fluorinated units upon immersion in water, while a second slower mechanism involves the migration of the oxyethylenic moieties to the polymer–water interface, dragged there by the covalently linked fluorinated tails and capable of forming hydrogen bonds with water. 

## 4. Conclusions

The new set of diblock copolymers presented a hydrophilic/hydrophobic character, which was designed by the ATRP synthesis to respond to contact with the outer environment. When the films were exposed to water, an unusual contraphilic behavior occurred. Modification of the polystyrene second block by the PEG-fluoralkyl chain carrying a longer PEG segment (x~12) caused such peculiar behavior. This in fact contrasts with the surface hydrophilicity of analogous block copolymers containing a PEG-fluoralkyl chain with a shorter PEG segment (x~5), for which a ‘normal’ significant increase in hydrophilicity upon immersion in water was reported.

The SEBS-based, two-layer films, containing the diblock copolymers as surface-active additives in the top layer, maintained the mechanical properties of the corresponding SEBS matrix, while the surface turned out to be much more lipophobic than the relative hydrocarbon matrix, owing to the presence of the fluorinated segments at the polymer–air interface. Nonetheless, the film surface became more enriched in fluorine at the polymer–water interface upon immersion in water, as expected of a distinct contraphilic surface. 

Overall, exhibiting bulk elastomeric properties, in addition to the surface hydrophobicity, low energy and responsiveness to water environment, fulfils principal prerequisites for the present two-layer films as candidates for use in antifouling/fouling-release coatings against marine biofouling. Their biological performance against model marine organisms, and their correlation with the film surface morphology and topography, will be the subjects of a forthcoming paper.

## Figures and Tables

**Table 1:** Experimental conditions for the synthesis and physical-chemical properties of block copolymers.

Polymer	SzA/S ^a)^	SzA (mol%)	SzA (wt %)	*M*_n_^b)^ (kg/mol)	*M*_n_^c)^ (kg/mol)	DP_n_^c)^ SzA	*Ð* ^b)^
S27SzA3	5	10	54	6.7	6.0	3	1.3
S27SzA11	20	29	81	12.6	14.5	11	1.4
S27SzA29	56	52	92	23.4	34.1	29	1.5

^a)^ Monomer/macroinitiator molar ratio in the feed. ^b)^ Number average molecular weight and dispersity by size exclusion chromatography (SEC). ^c)^ Number average molecular weight and degree of polymerization by proton nuclear magnetic resonance (^1^H NMR).

**Table 2:** Advancing and receding contact angles for S27SzAn block copolymers and the respective homopolymer.

Film	*θ*_a_ (°) ^a)^	*θ*_r_ (°) ^a)^	Δ (°) ^a)^	*θ*_a_ (°) ^b)^	*θ*_r_ (°) ^b)^	Δ (°) ^b)^
P(SzA) ^c)^	87	39	48	90 ± 0.8	38 ± 0.2	52
S27SzA3	96	65	31	98 ± 0.4	64 ± 0.3	34
S27SzA11	88	43	45	95 ± 0.6	41 ± 0.2	54
S27SzA29	87	39	48	93 ± 0.5	38 ± 0.1	55

^a)^ Advancing (*θ*_a_), receding (*θ*_r_) water contact angles and hysteresis of the film on 1st cycle. ^b)^ Average advancing (*θ*_a_) and receding (*θ*_r_) water contact angles and hysteresis on the wet film surface on 2nd and 3rd cycles. ^c)^ P(SzA) homopolymer (synthesized by radical polymerization initiated by 1 wt% azobisisobutyronitrile (AIBN) in anisole at 65 °C for 24 h).

**Table 3:** Static contact angles of the block copolymer films with water (*θ*_w_) and *n*-hexadecane (*θ*_h_).

Film	*θ*_w_ (°)	*θ*_h_ (°)
P(SzA)	84 ± 1	62 ± 1
S27SzA3	85 ± 1	64 ± 1
S27SzA11	86 ± 3	65 ± 1
S27SzA29	83 ± 1	65 ± 1

**Table 4:** Tensile test results for poly(styrene-*b*-(ethylene-*co*-butylene)-*b*-styrene) (SEBS)-based films and the corresponding SEBS (E30/E30-MA and E13/E13-MA) matrices.

Film	*ε*_max_^a)^ (%)	*σ*_max_^b)^ (MPa)	*E*^c)^ (MPa)
E30-S27SzA_0	513 ± 11	16.4 ± 0.8	3.6 ± 0.5
E30-S27SzA3_100	510 ± 13	16.0 ± 1.1	3.4 ± 0.5
E13- S27SzA_0	793 ± 51	10.2 ± 1.0	1.9 ± 0.0
E13- S27SzA3_100	930 ± 25	13.1 ± 0.9	1.8 ± 0.0
E13- S27SzA29_100	927 ± 52	12.3 ± 1.3	1.8 ± 0.0

^a)^ Maximum elongation at break. ^b)^ Maximum stress at break. ^c)^ Elastic modulus.

**Table 5:** Contact angles and surface tensions for the two-layer films and the corresponding SEBS bottom layer.

Film	*θ*_w_ (°) ^a)^	*θ*_h_ (°) ^a)^	*γ*_s_^OWK b)^ (mN/m)	*γ*_s_^d b)^ (mN/m)	*γ*_s_^p b)^ (mN/m)
E30-S27SzA_0	100 ± 1	28 ± 1	25.5	24.5	1.0
E30-S27SzA11_100	99 ± 1	67 ± 1	17.0	13.4	3.6
E30- S27SzA11_90	101 ± 1	65 ± 1	16.8	14.0	2.8
E30-S27SzA29_100	104 ± 1	65 ± 1	16.0	14.0	2.0
E30-S27SzA29_90	105 ± 1	64 ± 1	16.0	14.3	1.7
E13-S27SzA_0	99 ± 1	39 ± 1	23.4	21.8	1.6
E13-S27SzA3_100	96 ± 1	63 ± 2	18.9	14.6	4.3
E13-S27SzA11_100	102 ± 1	64 ± 1	16.7	14.2	2.5
E13-S27SzA11_90	101 ± 1	62 ± 1	17.5	14.9	2.6
E13-S27SzA29_100	104 ± 1	63 ± 1	16.5	14.6	1.9
E13-S27SzA29_90	105 ± 1	64 ± 1	16.0	14.3	1.7

^a)^ Measured with water and *n*-hexadecane. ^b)^ Calculated with the Owens–Wendt–Kaelble method; *γ*_s_^d^ dispersion component, *γ*_s_^p^ polar component.

**Table 6:** Experimental XPS atomic composition of the two-layer films before and after immersion in water for 7 days at different photoemission angles *φ.*

Film	*φ* (°)		Before Immersion			After Immersion		
			C (%)	O (%)	F (%)	C (%)	O (%)	F (%)
S27SzA11		Theor. ^a)^	67	15	18	67	15	18
	70		47.9	12.6	39.5	47.4	12.5	40.1
E13-S27SzA11_100	50		52.8	13.6	33.6	52.1	13.7	34.2
	20		60.0	15.3	24.7	59.2	15.4	25.4
S27SzA29		Theor. ^a)^	62	17	21	62	17	21
	70		47.1	13.3	39.6	46.4	13.4	40.2
E13-S27SzA29_100	50		50.8	15.1	34.1	50.1	15.6	34.3
	20		57.1	17.3	25.6	55.9	18.5	25.6
	70		49.9	13.1	37.0	49.9	12.9	37.2
E30-S27SzA11_90	50		53.4	14.7	31.9	53.4	13.6	33.0
	20		63.2	14.6	22.2	61.7	14.2	24.1
	70		48.0	13.5	38.5	46.4	13.1	40.5
E30-S27SzA29_90	50		51.0	15.1	33.9	50.5	14.6	34.9
	20		60.0	16.5	23.5	55.7	16.6	27.7

^a)^ Theoretical composition of the block copolymer.

## Supplementary Materials

The following are available online at https://www.mdpi.com/2073-4360/12/6/1236/s1. Figure S1: ^19^F NMR spectrum of S27SzA3. Figure S2: SEC traces of block copolymer S27SzAn and the corresponding macroinitiator S27. Figure S3: Reaction scheme of glass functionalization by GPS and SEBS-MA anchorage. Figure S4: Deconvolution of the C(1s) signal for the film E13-S27SzA11_100 at ***φ*** *=* 70° before immersion in water.
