# Supplementary material for: The Effect of Poly(ethylene glycol) (PEG) Length on the Wettability and Surface Chemistry of PEG-Fluoroalkyl-Modified Polystyrene Diblock Copolymers and Their Two-Layer Films with Elastomer Matrix
Source: Polymers (Basel). 2020 May 29;12(6):1236. doi: 10.3390/polym12061236 (PMC7361959; doi:10.3390/polym12061236)
Supplement: Supplementary file 1 [file polymers-12-01236-s001.pdf]

SUPPLEMENTARY INFORMATION

The Effect of Poly(ethylene glycol) (PEG) Length on the Wettability and Surface Chemistry of PEG-Fluoroalkyl-Modified Polystyrene Diblock Copolymers and Their Two-Layer Films with Elastomer Matrix

Elisa Guazzelli[1], Giancarlo Galli[1] , Elisa Martinelli[1*]

[1] Dipartimento di Chimica e Chimica Industriale and UdR Pisa INSTM, Università di Pisa, via Moruzzi 13, 56124 Pisa, Italy

*Corresponding author. Email: elisa.martinelli@unipi.it

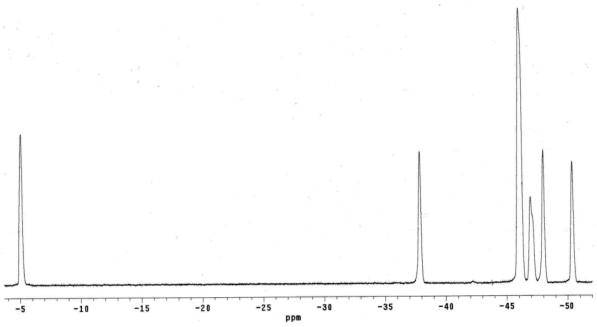

Figure S1. ^{19}F NMR spectrum of S27SzA3.

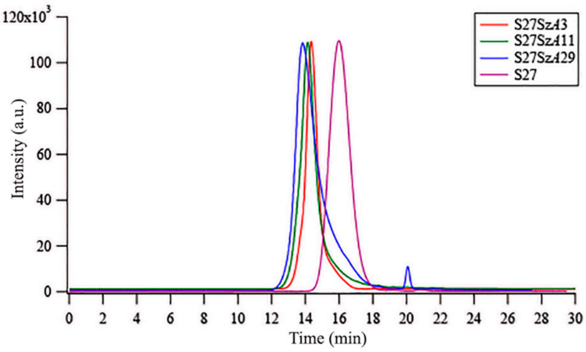

Figure S2. SEC traces of block copolymers S27SzAn and the corresponding macroinitiator S27.

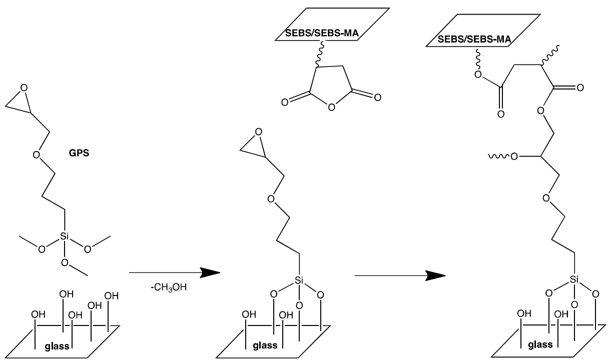

Figure S3. Reaction scheme of glass functionalization by GPS and SEBS-MA anchorage.

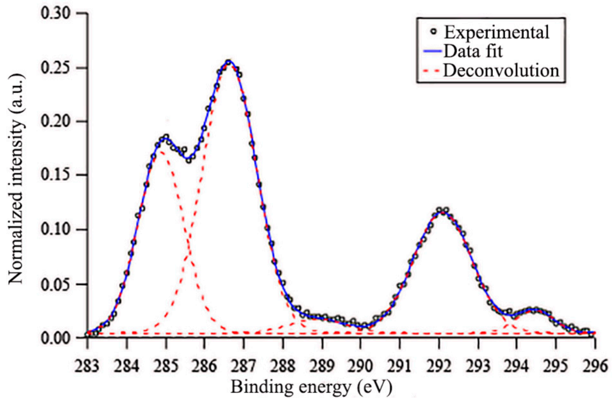

Figure S4. Deconvolution of the C(1s) signal for the film E13-S27SzA11_100 at ϕ = 70° before immersion in water.
